# Oregano Essential Oil Induces SOD1 and GSH Expression through Nrf2 Activation and Alleviates Hydrogen Peroxide-Induced Oxidative Damage in IPEC-J2 Cells

**DOI:** 10.1155/2016/5987183

**Published:** 2016-12-26

**Authors:** Yi Zou, Jun Wang, Jian Peng, Hongkui Wei

**Affiliations:** ^1^Department of Animal Nutrition and Feed Science, College of Animal Science and Technology, Huazhong Agricultural University, Wuhan 430070, China; ^2^The Cooperative Innovation Center for Sustainable Pig Production, Wuhan 430070, China

## Abstract

Oregano essential oil (OEO) has long been used to improve the health of animals, particularly their intestinal health. The health benefits of OEO are generally attributed to antioxidative actions, but the mechanisms remain unclear. Here, we investigate the antioxidative effects of OEO and their underlying molecular mechanisms in porcine small intestinal epithelial (IPEC-J2) cells. We found that OEO treatment prior to hydrogen peroxide (H_2_O_2_) exposure increased cell viability and prevented lactate dehydrogenase (LDH) release into the medium. H_2_O_2_-induced reactive oxygen species (ROS) and malondialdehyde (MDA) were remarkably suppressed by OEO. OEO dose-dependently increased mRNA and protein levels of the nuclear factor-erythroid 2-related factor-2 (Nrf2) target genes Cu/Zn-superoxide dismutase (SOD1) and g-glutamylcysteine ligase (GCLC, GLCM), as well as intracellular concentrations of SOD1 and glutathione. OEO also increased intranuclear expression of Nrf2 and the activity of an antioxidant response element reporter plasmid in IPEC-J2 cells. The OEO-induced expression of Nrf2-regulated genes and increased SOD1 and glutathione concentrations in IPEC-J2 cells were reduced by Nrf2 small interfering (si) RNAs, counteracting the protective effects of OEO against oxidative stress in IPEC-J2 cells. Our results suggest that OEO protects against H_2_O_2_-induced IPEC-J2 cell damage by inducing Nrf2 and related antioxidant enzymes.

## 1. Introduction

Oxidative stress is widely recognized as a state of imbalance between prooxidants and antioxidants. Excessive production of reactive oxygen species (ROS) and/or a deficiency of antioxidants result in endogenous oxidative stress [1]. The pathogenesis of various gastrointestinal diseases, such as peptic ulcers, gastrointestinal cancers, and inflammatory bowel disease, involves oxidative stress [2]. Oregano (*Origanum vulgare* L.) is an aromatic plant widely distributed throughout the Mediterranean area and Asia [3]. Oregano essential oil (OEO), a volatile oil, is a concentrate of natural plant products that contain the volatile aromatic compounds. This mixture of volatile aromatic compounds has several biological actions, including antimicrobial, anti-inflammatory, and antioxidative activities [4]. In recent studies, OEO has been demonstrated to possess antioxidant activity and prevent oxidative induced intestinal damage in animals [5–7]. These studies also suggest that OEO suppresses oxidative stress by inducing antioxidant enzymes, in addition to scavenging radicals. These studies also suggested that OEO suppress oxidative stress by inducing antioxidant enzymes, in addition to having radical-scavenging effects. However, the ability of OEO to induce antioxidant enzymes and the potential mechanisms in intestinal have not been reported.

Nuclear factor-erythroid 2-related factor-2 (Nrf2) is a transcription factor that plays a central role in cellular defenses against oxidative and electrophilic insults through the timely induction of antioxidant enzymes and related proteins [8]. Under normal conditions, Nrf2 is bound to Kelch-like ECH-associated protein 1 (Keap1) and is ubiquitinated and targeted for degradation [9]. Shear stress, dietary antioxidants, and other physiological stimuli that disrupt Keap1-Nrf2 interactions allow nuclear accumulation of Nrf2, resulting in the transcription of antioxidant enzymes such as g-glutamyl cysteine ligase (GCL) and Cu/Zn-superoxide dismutase (SOD1) [10–12]. GCL is a heterodimer consisting of catalytic (GCLC) and modifier (GCLM) subunits, both of which are products of Nrf2 target genes. GCL has been extensively investigated for its ability to regulate the synthesis of glutathione (GSH), the most abundant natural cellular antioxidant, which plays an essential role in maintaining the cellular redox state [13]. SOD plays an important role in the defense mechanisms of biological cells exposed to oxygen [14], catalyzing the dismutation of superoxide anion radical (O_2_^−^) into an oxygen molecule and a hydrogen peroxide (H_2_O_2_) [15]. This reaction is recognized as an antioxidant system that protects cells from superoxide toxicity. Cu/Zn-SOD (SOD1) exists in the cytoplasm, lysosomes, and nuclear compartments of mammalian cells [16].

OEO has been shown to have antioxidant properties in in vivo studies, but its antioxidant role is not well understood. The objectives of this study were to investigate the oxidative damaging effects of H_2_O_2_ on growth of porcine small intestinal epithelial (IPEC-J2) cells in vitro, determine whether OEO has an antioxidative effect (restoring the cell redox state during oxidative stress) on IPEC-J2 cells, and, if OEO does have an antioxidative effect, to elucidate the underlying molecular mechanisms.

## 2. Materials and Methods

### 2.1. Chemicals and Regents

Dulbecco's Modified Eagle Medium (DMEM) and fetal bovine serum (FBS) were purchased from GIBCO BRL (Grand Island, NY, USA). OEO was obtained from Meritech Bioengineering Co. Ltd. (Guangzhou, China), and its composition is shown in Table 1. Typical chromatogram of oregano essential oil is shown in Supplementary Material available online at http://dx.doi.org/10.1155/2016/5987183. H_2_O_2_ solution, 3-(4,5-dimethyl-2-thiazolyl)-2,5-diphenyl-2H-tetrazolium bromide (MTT), 2-(4-amidinophenyl)-6-indolecarbamidine dihydrochloride (DAPI), and 2′,7′-dichlorofluorescein-diacetate (DCFH_2_-DA) were obtained from Sigma-Aldrich (St. Louis, MO, USA). Antibodies against Nrf2, Keap1, and *β*-actin were obtained from Cell Signaling Technology (Beverly, MA, USA). Antibodies against GCLM, *γ*-GCLC, and SOD1 were obtained from Affinity Biosciences (Columbus, OH, USA). The antibody against proliferating cell nuclear antigen (PCNA) was obtained from BD Transduction Laboratories (San Diego, CA, USA). The HRP-conjugated secondary antibodies, anti-rabbit IgG and anti-mouse IgG, were purchased from Jackson ImmunoResearch (West Grove, PA, USA). IPEC-J2 cells were obtained from the Cell Bank of Type Culture Collection of the Chinese Academy of Sciences Institution (Chinese Academy of Sciences, China) and cultured at 37°C in a 5% CO_2_ atmosphere in DMEM containing 10% FBS. OEO was dissolved in the serum-containing medium to achieve the final desired concentration.

### 2.2. MTT Assay

Cell viability was monitored using an MTT colorimetric assay. IPEC-J2 cells (2 × 10^4^ per well in a 96-well plate) were incubated with or without OEO and H_2_O_2_. Cells were washed with phosphate-buffered saline (PBS) and then incubated with 10 *μ*L of 5 mg/mL MTT in PBS for 4 h. The supernatant was removed and the culture resuspended with 150 *μ*L of isopropanol to dissolve MTT formazan. The absorbance was measured at 490 nm using a spectrophotometer (Biomate 5, Thermo Electron Corporation, Rochester, NY, USA). The effect of OEO and H_2_O_2_ on cell viability was assessed as the percentage of viable cells compared with vehicle-treated control cells, which were arbitrarily assigned a viability of 100%.

### 2.3. Lactate Dehydrogenase (LDH) Release Assay

The activity of LDH released into the culture medium through damaged membranes was measured spectrophotometrically using an LDH cytotoxicity assay kit (Nanjing Jiancheng Institute of Bioengineering, Nanjing, China) according to the manufacturer's protocol. The method is based on the LDH-catalyzed reduction of pyruvate to lactate by an equimolar amount of NADH. NADH formation is detected by the release of a chromogen that emits at 490 nm. IPEC-J2 cells (2 × 10^4^ per well in a 96-well plate) were pretreated with different concentrations of OEO and then exposed to H_2_O_2_. The absorbance was measured with an ELISA microplate reader, and any decrease in absorbance was monitored.

### 2.4. Measurement of ROS Generation

The intracellular accumulation of ROS was detected by fluorescence microscopy using DCFH_2_-DA. IPEC-J2 cells (1 × 10^5^ cells/well in a 6-well plate) were cultured in DMEM supplemented with 10% FBS; culture medium was renewed when cells reached 80% confluence. After OEO pretreatment (90% confluence) and H_2_O_2_ incubation, cell culture media were removed, and cells were washed with PBS and incubated with 10 *μ*M DCFH_2_-DA in fresh culture medium at 37°C for 30 min. The acetate groups on DCFH_2_-DA were removed by an intracellular esterase, trapping the probe inside IPEC-J2 cells. Intracellular ROS, as indicated by DCF fluorescence, was measured with a fluorescence microscope (ECLIOSE Ti, Nikon, Japan). The fluorescence intensity under each condition was quantified on a FACSCalibur flow cytometer (BD Biosciences, San Diego, CA, USA) using excitation and emission filters of 488 and 530 nm, respectively. Levels of ROS were measured in the supernatant of IPEC-J2 cells by chemiluminescence assay using luminol (5-amino-2,3-dihydro-1,4-phthalazinedione, Sigma, St. Louis, MO, USA) as described in our previous study [17]. The results were expressed as relative light units (RLU).

### 2.5. Assessment of Lipid Peroxidation

IPEC-J2 cells (1 × 10^5^ per well in a 6-well plate) were incubated with or without OEO and H_2_O_2_. The content of malondialdehyde (MDA) in IPEC-J2 cells was assayed using colorimetric methods with a spectrophotometer (Biomate 5). The assays were conducted with kits purchased from Nanjing Jiancheng Institute of Bioengineering according to the manufacturer's instructions. IPEC-J2 cells were washed twice with cold PBS and harvested in 5% metaphosphoric acid using a cell scraper. Harvested cells were lysed by sonication on ice, and the supernatant of the centrifugate (1300 ×g at 4°C for 15 min) was collected. Samples were vortexed with 8.1% sodium dodecyl sulfate, 20% acetic acid, and 0.8% 2-thiobarbituric acid and incubated for 1 h at 95°C before butanol-pyridine 15 : 1 (v/v) was added. The mixture was shaken for 10 min and then centrifuged (1300 ×g at 4°C). The butanol-pyridine layer was measured fluorometrically at 552 nm.

### 2.6. Quantitative PCR

IPEC-J2 cells (5 × 10^4^ per well in a 24-well plate) were incubated with or without OEO. Total RNA was extracted from samples of IPEC-J2 cells using Trizol reagent (Invitrogen, Carlsbad, CA, USA) according to the manufacturer's instructions. Total RNA was determined using a NanoDrop® ND-1000 spectrophotometer (NanoDrop Technologies, Wilmington, DE, USA). For cDNA synthesis, 2.5 *μ*g of RNA was reverse-transcribed using the Prime Script RT reagent kit (Takara, Otsu, Japan) according to the manufacturer's protocol. The primers used in this study, synthesized according to our previous protocols or designed with Primer 5.0 according to pig gene sequences, were as follows: Nrf2 5′-GAAAGCCCAGTCTTCATTGC-3′ (sense), 5′-TTGGAACCGTGCTAGTCTCA-3′ (antisense); SOD1 5′-ACCTGG-GCAATGTG-ACTG-3′ (sense), 5′-TCCAGCATTTCCCGTCT-3′ (antisense); CAT 5′-AACTGTCCCTTCCGTGCTA-3′ (sense), 5′-CCTGGGTGACATTATC-TTCG-3′ (antisense); GCLC 5′-CATTGCGACACACTGGAGAC-3′ (sense), 5′-CAAACCA-TCCTACCCTTTGG-3′ (antisense); GCLM 5′-ATTGTGCAGAGAGCCTGGTT-3′ (sense), and 5′-ACAATACAACGGTTCAGGTGAGT-3′ (antisense). Real-time PCR was performed as described in our previous study [6]. The relative expression of genes in the treatment group was normalized to the values of the control group.

### 2.7. Western Blotting Analysis

IPEC-2 cells (1 × 10^6^ cells per well in a 6-well plate) were collected and whole-cell lysates were prepared in RIPA buffer containing protease inhibitors or phosphatase inhibitors (Thermo Scientific, Rockford, IL, USA). Nuclear lysates were prepared using a nuclear/cytosol fractionation kit (BestBio, Shanghai, China), according to the manufacturer's protocol. They were then centrifuged at 12,000 ×g at 4°C for 10 min, and the supernatants were collected for assay. Protein concentrations were determined using a standard BCA protein assay, and 30 *μ*g protein was loaded per sample/lane and separated on SDS-PAGE. Protein samples were then electrophoretically transferred to nitrocellulose membranes, which were blocked in TBST (5% nonfat milk, 10 mM Tris, 150 mM NaCl, and 0.05% Tween-20) for 2 h. The blots were then incubated with one of the following primary antibodies at 4°C overnight: anti-Nrf2 (1 : 1000), anti-Keap1 (1 : 1000), anti-GCLM (1 : 1000), anti-GCLC (1 : 1000), anti-SOD1 (1 : 1000), anti-PCNA (1 : 5000), and anti-actin (1 : 1000). After three washes with Tris-buffered saline containing 0.1% Tween 20, blots were incubated with an HRP-conjugated secondary antibody, anti-rabbit IgG (1 : 15000), or anti-mouse IgG (1 : 15000), for 2 h, and then were washed again. Chemiluminescence detection was performed using ECL reagent (Thermo Scientific) according to the manufacturer's instructions. Specific bands were detected, analyzed, and quantified by Image J Software (NIH, Bethesda, MD, USA).

### 2.8. Intracellular GSH and SOD1 Measurement

For these assays, IPEC-J2 cell number was optimized at 1 × 10^6^ per well in 6-well plate. Cells were washed twice with cold PBS and harvested in 5% metaphosphoric acid using a cell scraper. Harvested cells were lysed by sonication on ice, and the supernatant of the centrifugate (1300 ×g at 4°C for 15 min) was collected. GSH levels were measured colorimetrically using a GSH assay kit (Nanjing Jiancheng Institute of Bioengineering) according to the manufacturer's protocol and normalized to the protein concentration in the lysate. This assay is based on the spectrophotometric measurement of 5,5′-dithiobis(2-nitrobenzoic acid) (DTNB), the product of a reaction with GSH. The yellow derivative 5′-thio-2-nitrobenzoic acid (TNB) was measured by detecting absorbance at 412 nm using a microplate reader. SOD1 activity was determined by measuring inhibition of the reduction of the water-soluble tetrazolium salt, WST-8 (2-(2-methoxy-4-nitrophenyl)-3-(4-nitrophenyl)-5-(2,4-disulfophenyl)-2H-tetrazolium, monosodium salt), which produces a water-soluble formazan dye upon reduction with O_2_^−•^. Determinations were performed with a Superoxide Dismutase Assay Kit (Beyotime Biotechnology, ShangHai, China) specific to SOD1.

### 2.9. Nrf2 Immunofluorescence

IPEC-J2 cells (1 × 10^5^ per well) were seeded on diameter glass coverslips coated with poly-L-lysine in a 6-well plate. Cells were incubated with or without 10 *μ*g/mL of OEO for 24 h. After being washed with cold PBS for three times, cells were fixed with 4% paraformaldehyde for 15 min, permeabilized with Triton X-100 (0.1%) for 15 min, and blocked with bovine serum albumin (1%) for 30 min. After being washed with PBS, cells were incubated overnight with Nrf2 antibody diluted 1 : 100 at 4°C, followed by incubation with Alexa Fluor 568-conjugated secondary antibody for 1 h. DAPI staining was performed to define nuclear regions. The fluorescence images were captured by laser scanning confocal microscopy (Eclipse TI, Nikon, Tokyo, Japan).

### 2.10. Luciferase Reporter Assay

The antioxidant response element (ARE) luciferase reporter plasmid (pGL6 (pARE-luc)) was obtained from Beyotime Biotechnology. IPEC-J2 cells were plated into 6-well plates (1 × 10^5^ cells/well) and cultured for 24 h. Cells were cotransfected with 0.5 *μ*g of luciferase expression plasmid and 1 *μ*g of PRL-TK plasmid (Promega, Madison, USA) as a normalization reference for transfection efficiency using SuperFect transfection reagent (Qiagen, Valencia, CA, USA) for 24 h and then stimulated with OEO 12 h. Cells were harvested, and firefly and Renilla luciferase activities were determined using a Dual-Luciferase Reporter assay system (Progema). The ratio (reporter/control luciferase activity) obtained from control cell lysate was set at one.

### 2.11. SiRNA Transfection in IPEC-J2 Cells

The double-stranded pig Nrf2 siRNA 5′-GCCCAUUGAUCUCUCUGAUTT-3′ (sense) and 5′-AUCAGAGAGAUCAAUGGGCTT-3′ (antisense) were synthesized by GenePharma Co. Ltd. (Shanghai, China). IPEC-J2 cells were plated into 6-well plates (1 × 10^5^ cells/well) and cultured for 24 h. Cells were transfected with 100 nM siRNA against Nrf2 or scrambled duplex using LipofectAMINE 2000 (Invitrogen). After 24-hour incubation, fresh medium was added and the cells were cultured for another 24 h before treatment with OEO.

### 2.12. Statistical Analyses

Statistical analysis was performed using Prism software (Prism 5.0; GraphPad Software, La Jolla, CA, USA). Data were analyzed by Student's *t*-test, Student-Newman-Keuls multiple-comparison test, or two-way ANOVA using SAS v 8.2 (SAS Inst. Inc., Cary, NC, USA). Student's *t*-test was used for comparisons of two groups, Student-Newman-Keuls multiple-comparison test was used for comparisons among groups, and OEO concentration and Nrf2 siRNA were used as factors in the two-way ANOVA. Values are presented as means ± standard error of the mean (SEM), and those at *P* < 0.05 were considered significant.

## 3. Results

### 3.1. Preventive Effect of OEO on Oxidative Stress-Induced Toxicity in IPEC-J2 Cells

**Figure 1:**
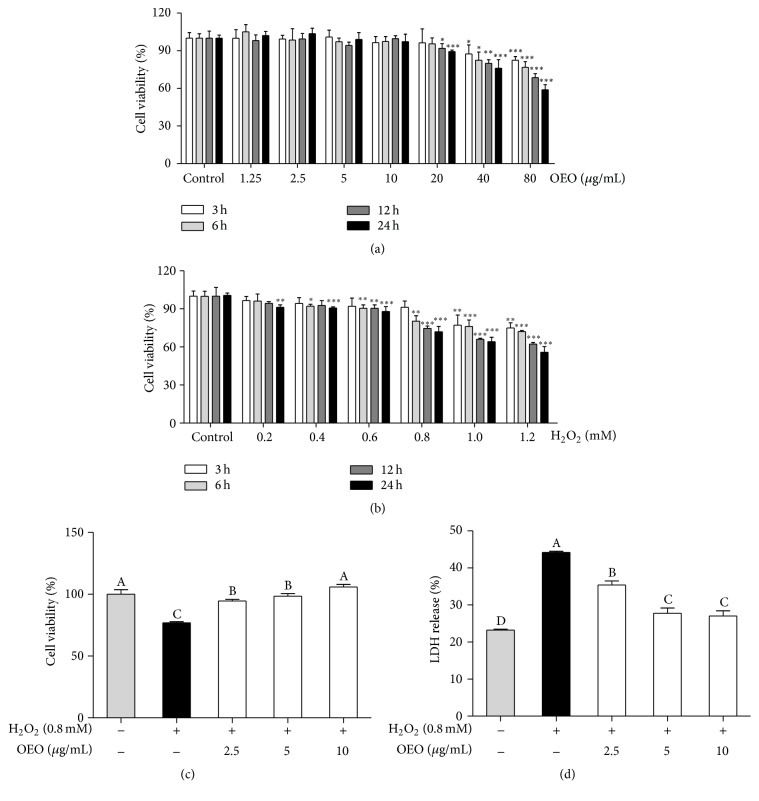
Oregano essential oil (OEO) inhibits hydrogen peroxide- (H_2_O_2_-) induced cytotoxicity in IPEC-J2 cells. (a) IPEC-J2 cells were incubated with OEO (1.25–80 *μ*g/mL) for 3–24 h. Cell viability was determined by MTT assay. (b) IPEC-J2 cells were incubated with H_2_O_2_ (0.2–1.2 mmol) for 3–24 h. Cell viability was determined by MTT assay. (c) IPEC-J2 cells were incubated with OEO (2.5–10 *μ*g/mL) for 24 h and then incubated with 0.8 mM H_2_O_2_ for 24 h. The protective effects of OEO were evaluated by MTT assay. (d) IPEC-J2 cells were incubated with OEO (2.5–10 *μ*g/mL) for 24 h and then incubated with 0.8 mM H_2_O_2_ for 24 h. The protective effects of OEO were evaluated by lactate dehydrogenase (LDH) release assay. Values represent means ± SEM, *n* = 3. ^*∗*^ *P* < 0.05, ^*∗∗*^ *P* < 0.01, and ^*∗∗∗*^ *P* < 0.001, compared with untreated control cells; means without a common letter differ, *P* < 0.05.

The cytotoxic effects of OEO on IPEC-J2 cells were evaluated by MTT assay. The percentage of viable cells was compared with that of the control cells. Cells were treated with concentrations of OEO ranging from 1.25 to 80 *μ*g/mL for 3, 6, 12, or 24 h, to clarify the role of the time of exposure. Low doses of OEO (1.25–10 *μ*g/mL) did not cause cytotoxic effects at any duration of exposure. OEO doses of 20–80 *μ*g/mL for 24 h significantly reduced cell viability compared with untreated control cells (Figure 1(a)). Based on these cell viability data, subsequent experiments were performed using concentrations of OEO ≤10 *μ*g/mL. Cells were treated with concentrations of H_2_O_2_ ranging from 0.2 to 1.2 mM for 3, 6, 12, or 24 h to clarify the role of the time of exposure. The results of cell exposure to H_2_O_2_ are shown in Figure 1(b). The viability of cells treated with 0.8 mM H_2_O_2_ for 24 h was approximately 70% of that of the control cells. Therefore, treatment with 0.8 mM H_2_O_2_ 24 h was selected for subsequent experiments.

Pretreatment of IPEC-J2 cells with OEO significantly improved cell viability in the presence of H_2_O_2_ in a dose-dependent manner (Figure 1(c)). To further explore the protective effect of OEO, the release of LDH, an indicator of cell injury, was also examined in H_2_O_2_-exposed IPEC-J2 cells. The results showed that pretreatment with OEO attenuated the H_2_O_2_-induced increase in LDH, affirming its protective role (Figure 1(d)).

### 3.2. OEO Inhibits Oxidative Stress-Induced ROS and MDA Production in IPEC-J2 Cells

**Figure 2:**
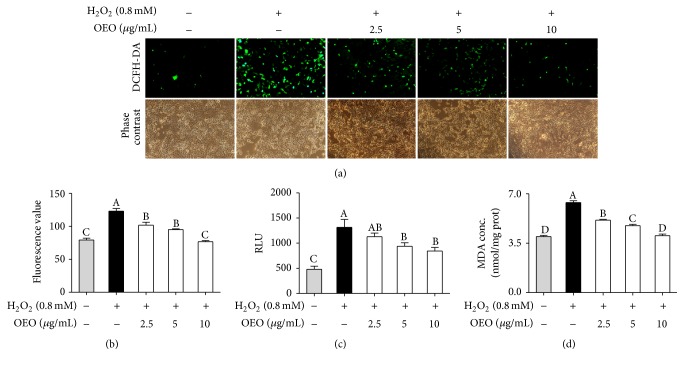
Oregano essential oil (OEO) inhibits hydrogen peroxide- (H_2_O_2_-) induced reactive oxygen species (ROS) and malondialdehyde (MDA) production in IPEC-J2 cells. (a) Cells were pretreated with OEO (2.5–10 *μ*g/mL) for 24 h and then incubated with 0.8 mM H_2_O_2_ for 24 h. Intracellular ROS levels were indicated by DCF fluorescence and detected by fluorescence microscopy (200x magnification). (b) Cells were pretreated with OEO (2.5–10 *μ*g/mL) for 24 h and then incubated with 0.8 mM H_2_O_2_ for 24 h. The fluorescence intensity of DCF-stained cells was quantified by flow cytometer. (c) Cells were pretreated with OEO (2.5–10 *μ*g/mL) for 24 h and then incubated with 0.8 mM H_2_O_2_ for 24 h. The levels of ROS in culture supernatant were measured by chemiluminescence. Chemiluminometric counts were obtained at 0.05 sec intervals for 5 sec, and the results were expressed as areas under the curve of relative light units (RLU) for 5 sec. (d) Cells were pretreated with OEO (2.5–10 *μ*g/mL) for 24 h and then incubated with 0.8 mM H_2_O_2_ for 24 h. The intracellular MDA levels were measured. Values represent means ± SEM, *n* = 3. Means without a common letter differ, *P* < 0.05.

H_2_O_2_-induced intracellular ROS production in IPEC-J2 cells was monitored by fluorescence microscopic analysis using DCFH_2_-DA as a fluorescent probe. IPEC-J2 cells exposed to H_2_O_2_ (0.8 mM) for 24 h showed a significant increase in fluorescence, which is proportionate to the amount of ROS generated (Figure 2(a)). This ROS induction was substantially inhibited by OEO pretreatment (2.5–10 *μ*g/mL) (Figure 2(b)). Levels of ROS were also measured in the supernatant of IPEC-J2 cells by chemiluminescence assay using luminol as a probe. Pretreatment with OEO inhibited ROS levels in IPEC-J2 cell supernatants in the presence of H_2_O_2_ in a dose-dependent manner (Figure 2(c)). Thus, the protective effects of OEO in IPEC-J2 cells are further evidenced by its inhibition of excessive ROS production.

MDA is the end product of lipoperoxidation and is considered to be an excellent indicator of oxidative stress [18]. To investigate the effect of OEO on cell lipid peroxidation in cultured IPEC-J2 cells, levels of MDA were measured in IPEC-J2 cells using the TBARS assay. As shown in Figure 2(d), MDA levels in IPEC-J2 cells were markedly increased in response to H_2_O_2_ compared with the control group, whereas pretreatment with OEO significantly inhibited lipid peroxidation in a dose-dependent manner.

### 3.3. OEO Increases Nrf2-Regulated Antioxidant Enzyme Expression

**Figure 3:**
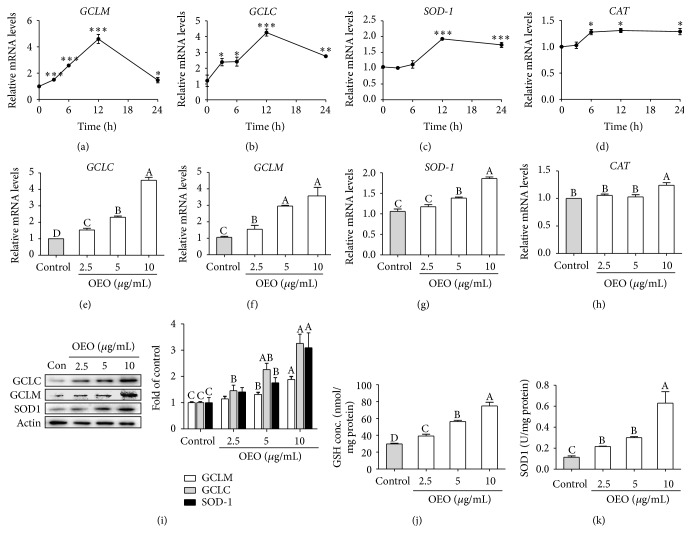
Effects of oregano essential oil (OEO) on g-glutamylcysteine ligase (GCLM, GCLC), and Cu/Zn-superoxide dismutase (SOD1) expression in IPEC-J2 cells. (a–d) IPEC-J2 cells were incubated with OEO 10 *μ*g/mL for 3–24 h. (e–h) IPEC-J2 cells were incubated with OEO (2.5–10 *μ*g/mL) for 12 h. Relative mRNA expression levels were measured quantitatively using real-time RT-PCR. The results were normalized to *β*-actin and expressed as fold increase over control. (i) IPEC-J2 cells were incubated with OEO (2.5–10 *μ*g/mL) for 24 h. GCLC, GCLM, and SOD1 were estimated by western blot. (j) IPEC-J2 cells were incubated with OEO (2.5–10 *μ*g/mL) for 24 h, and intracellular glutathione (GSH) content was determined. (k) IPEC-J2 cells were incubated with OEO (2.5–10 *μ*g/mL) for 24 h, and intracellular SOD1 content was determined. Values represent means ± SEM, *n* = 3. ^*∗*^ *P* < 0.05, ^*∗∗*^ *P* < 0.01, and ^*∗∗∗*^ *P* < 0.001, compared with untreated control cells. Means without a common letter differ, *P* < 0.05.

We predicted that the protective effects of OEO against oxidative stress occurred as a result of the induction of antioxidant genes. The expression of antioxidant genes SOD1, catalase (CAT), GCLM, and GCLC under different conditions was analyzed. IPEC-J2 cells were treated with 10 *μ*g/mL of OEO for 3, 6, 12, or 24 h. OEO caused a maximal increase in SOD1, CAT, GCLM, and GCLC mRNA expression levels at 12 h (Figures 3(a), 3(b), 3(c), and 3(d)). Treatment with OEO at 2.5–10 *μ*g/mL for 12 h dose-dependently increased GCLM, GCLC, and SOD1 mRNA expression levels (Figures 3(e), 3(f), 3(g), and 3(h)), and pretreatment with 10 *μ*g/mL of OEO upregulated CAT gene expression by 1.24-fold, compared with the nontreatment group. OEO also dose-dependently increased intracellular protein concentrations of GCLM, GCLC, and SOD1 (Figure 3(i)) and increased intracellular GSH (Figure 3(j)) and SOD1 (Figure 3(k)) protein concentrations in a dose-dependent manner.

### 3.4. Activation of Nrf2 by OEO

**Figure 4:**
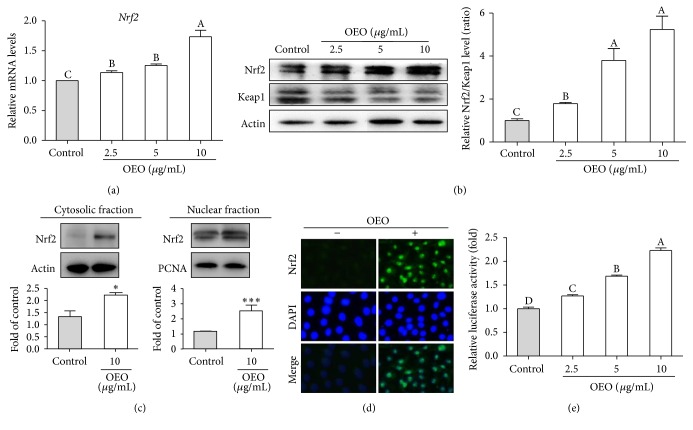
Effects of oregano essential oil (OEO) on nuclear factor-erythroid 2-related factor-2 (Nrf2) activation in IPEC-J2 cells. (a) IPEC-J2 cells were incubated with OEO (2.5–10 *μ*g/mL) for 12 h. Relative Nrf2 mRNA expression levels were measured quantitatively using real-time RT-PCR. (b) IPEC-J2 cells were incubated with OEO 10 *μ*g/mL for 24 h and then Nrf2 and Kelch-like ECH-associated protein 1 (Keap1) were estimated in IPEC-J2 cells. (c) IPEC-J2 cells were incubated with OEO 10 *μ*g/mL for 24 h; then Nrf2 was estimated in the cytosolic and nuclear fractions. Western blot results show the effects of OEO on protein levels of Nrf2 in the cytosolic and nuclear fractions. (d) Localization of endogenous Nrf2. IPEC-J2 cells were incubated with OEO 10 *μ*g/mL for 24 h and then subjected to immunohistochemical staining with antibodies specific for Nrf2 followed by incubation with Alexa Fluor 568-conjugated secondary antibodies. For each condition, an image of the cell nucleus stained with DAPI specific for nuclear proteins is also presented. The immunohistochemical staining was observed using fluorescence microscopy (200x magnification). (e) IPEC-J2 cells were incubated with OEO 10 *μ*g/mL for 24 h; then levels of Nrf2 and Keap1 were estimated in IPEC-J2 cells. (e) IPEC-J2 cells were cotransfected with a reporter plasmid (pGL6) and a control plasmid (pRL-TK). After transfection, IPEC-J2 cells were incubated with OEO (2.5–10 *μ*g/mL) for 12 h; then luciferase activity was measured. Values represent means ± SEM, *n* = 3. ^*∗*^ *P* < 0.05 and ^*∗∗∗*^ *P* < 0.001, compared with untreated control cells. Means without a common letter differ, *P* < 0.05.

Nrf2 is a major transcription factor involved in cellular protection against oxidative stress through ARE-mediated induction of antioxidant enzymes [19]. As shown in Figure 4(a), treatment with OEO at 2.5–10 *μ*g/mL for 12 h dose-dependently increased Nrf2 mRNA expression levels. Western blots demonstrated that OEO incubation (2.5–10 *μ*g/mL) for 24 h increased the ratio of Nrf2 to Keap1 in a semi-dose-dependent manner (Figure 4(b)). To investigate OEO-mediated nuclear translocation of Nrf2, the amount of cytosolic and nuclear Nrf2 protein was determined in the respective fractions of IPEC-J2 cells. The results showed increased nuclear Nrf2 and decreased cytosolic Nrf2 with 10 *μ*g/mL of OEO alone (Figure 4(c)). For further confirmation, nuclear import of Nrf2 in control and treated cells was monitored by immunofluorescence. As illustrated in Figure 4(d), Nrf2 was located in the cytoplasm of control cells, but the amount of nuclear Nrf2 increased after OEO treatment (10 *μ*g/mL). To evaluate the involvement of Nrf2 in OEO-induced ARE activity, IPEC-J2 cells were transfected with Nrf2 expression plasmids and OEO enhanced the activation of ARE in a dose-dependent manner (Figure 4(e)).

### 3.5. Antioxidant Gene Induction by OEO is Dependent on Nrf2

**Figure 5:**
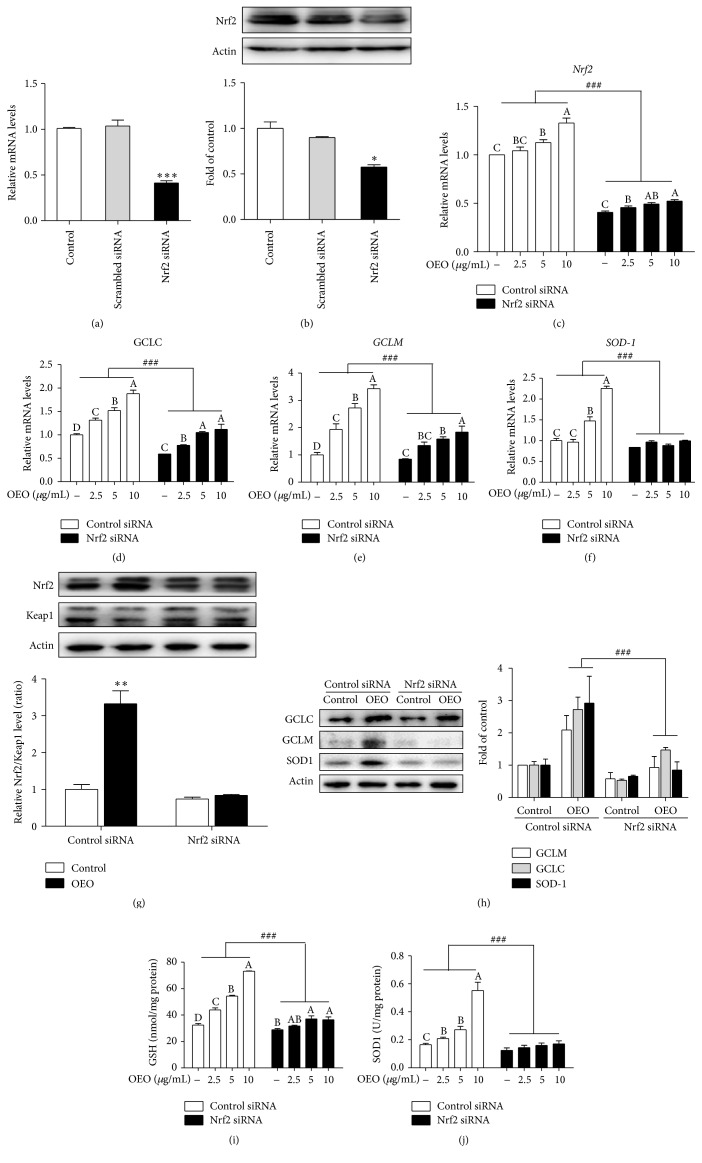
Effects of oregano essential oil (OEO) on g-glutamylcysteine ligase (GCLM, GCLC) and Cu/Zn-superoxide dismutase (SOD1) expression in nuclear factor-erythroid 2-related factor-2 (Nrf2) knockdown IPEC-J2 cells. (a, b) IPEC-J2 cells were treated with Nrf2 siRNA or control siRNA and incubated for 24 h. (a) Nrf2 mRNA expression was quantitated and normalized as for Figure 3(a). (b) Whole-cell lysates were subjected to western blotting. (c–f) IPEC-J2 cells were transfected with siRNA against Nrf2 or control siRNA. After 24 h, the cells were incubated with OEO (2.5–10 *μ*g/mL) for an additional 12 h. Nrf2, GCLC, GCLM, and SOD1 mRNA expression levels were measured quantitatively using real-time RT-PCR. (g-h) IPEC-J2 cells were transfected with siRNA against Nrf2 or control siRNA. After 24 h, the cells were incubated with OEO 10 *μ*g/mL for an additional 24 h. Nrf2, Kelch-like ECH-associated protein 1 (Keap1), GCLC, GCLM, and SOD1 were estimated by western blot. (i) Effect of OEO on intracellular glutathione (GSH) content in Nrf2 knockdown IPEC-J2 cells. OEO (2.5–10 *μ*g/mL) was applied as described for Figure 3(j), and intracellular GSH content was measured. (j) Effect of OEO on intracellular SOD1 content in Nrf2 knockdown IPEC-J2 cells. OEO (2.5–10 *μ*g/mL) was applied as described for Figure 3(k), and intracellular SOD1 content was measured. Values represent means ± SEM, *n* = 3. ^*∗*^ *P* < 0.05, ^*∗∗*^ *P* < 0.01, and ^*∗∗∗*^ *P* < 0.001, compared with control cells treated with control siRNA; ^###^ *P* < 0.001, compared with corresponding cells treated with control siRNA. Means without a common letter differ, *P* < 0.05.

Nrf2 was downregulated by treatment with Nrf2 siRNA, as shown in Figures 5(a) and 5(b). Treatment with Nrf2 siRNA approximately reduced Nrf2 mRNA expression and protein expression by 53% and 47%, respectively. The increasing expressions of Nrf2, SOD1, GCLM, and GCLC mRNA caused by the OEO (2.5–10 *μ*g/mL) were suppressed by Nrf2 siRNA (Figures 5(c), 5(d), 5(e), and 5(f)). As shown in Figure 5(g), Nrf2 siRNA reduced baseline Nrf2 protein as well as OEO-induced Nrf2 protein. This finding coincided with the greater reduction of the OEO-induced (10 *μ*g/mL) Nrf2, SOD1, GCLM, and GCLC protein expression levels (Figure 5(h)). Similarly, Nrf2 siRNA also reduced the intracellular GSH and SOD1 induced by OEO (Figures 5(i) and 5(j)). It is worth noting, however, that treatment with OEO still increased GCLM, GCLC, and Nrf2 mRNA expression levels and intracellular GSH under conditions of Nrf2 suppression (Figures 5(c), 5(d), 5(e), and 5(i)).

### 3.6. Cytoprotection Against Oxidative Stress by OEO is Dependent on Nrf2

**Figure 6:**
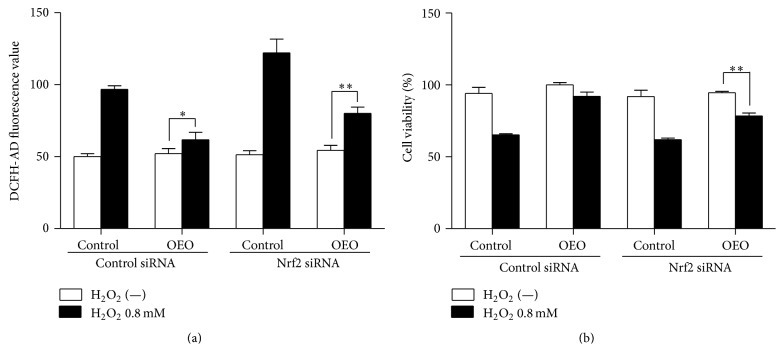
Nuclear factor-erythroid 2-related factor-2 (Nrf2) siRNA diminishes the protective effects of IPEC-J2 cells. IPEC-J2 cells were treated with Nrf2 siRNA or control siRNA and incubated for 24 h. Cells were incubated with oregano essential oil (OEO, 10 *μ*g/mL for 24 h) and then incubated with H_2_O_2_ (0.8 mM) for 24 h. (a) Cell viability was determined by MTT assay. (b) Intracellular reactive oxygen species (ROS) levels were measured by DCF fluorescence microscopy. Values represent means ± SEM, *n* = 3, significant at ^*∗*^ *P* < 0.05, ^*∗∗*^ *P* < 0.01.

The role of Nrf2 in the antioxidative effect of OEO against H_2_O_2_-induced cell toxicity was investigated in Nrf2 knockdown IPEC-J2 cells. OEO (10 *μ*g/mL) suppressed H_2_O_2_-induced cell toxicity in cells treated with control siRNA, but the protective effects were reduced in Nrf2 knockdown cells (Figure 6(a)). Furthermore, the reduction in intracellular ROS induced by OEO was inhibited by knockdown of Nrf2 (Figure 6(b)), indicating that this antioxidative effect of OEO was mediated by Nrf2.

## 4. Discussion

The gastrointestinal tract is prone to ROS attack as it is accessed by entities from the outside environment, with resident immune cells and intestinal flora as well as dietary factors as potential sources of ROS [2]. Oxidative stress has been widely implicated in intestinal injury under both in vivo [20, 21] and in vitro conditions [22, 23]. Recent studies have shown that OEO reduces oxidative stress and increases antioxidant enzymes in the intestines of animals [5, 7]. Furthermore, in a previous study in our laboratory, we observed that OEO increases antioxidant enzyme expression and reduces ROS levels in the intestine of rats in a model of oxidative stress [6]. However, the detailed molecular mechanisms by which OEO improves antioxidant status are unknown. To elucidate the mechanisms by which these antioxidant effects are exerted, here we assessed the effects of OEO on antioxidant enzymes and molecules in porcine IPEC-J2 intestinal epithelial cells. The OEO used in the present study contains volatile, natural, complex compounds characterized by a strong odor, formed by* Origanum vulgare* L as secondary metabolites. The main components of the OEO were thymol and carvacrol, while their biogenetic precursors, *ρ*-Cymene and *γ*-Terpinene, were the most abundant monoterpenes (Table 1). Importantly, the OEO concentrations (2.5–10 *μ*g/mL) used in the present study were noncytotoxic and very low. Favorable pharmacological properties of OEO have been demonstrated in several studies at similarly low concentrations. For instance, treatment of oxidized low density lipoprotein-activated THP-1 macrophages with OEO (10–30 *μ*g/mL) results in an overall reduction in proinflammatory cytokine release [24]. Likewise, antioxidative effects of OEO components thymol and carvacrol (8 to 30 *μ*g/mL) have been demonstrated in Caco-2 cells [25]. To our knowledge, this study is the first to demonstrate that OEO increases antioxidant enzyme expression levels and prevents oxidative stress-induced cell death through activation of Nrf2.

H_2_O_2_ is often used in studies of redox-regulated processes because it is relatively stable compared with other ROS [26]. It has been well documented that exposure to H_2_O_2_ triggers the rapid generation of ROS in various cell types [27–29], including IPEC-J2 cells [23]. Accumulation of ROS and impairment of the antioxidant defense system by H_2_O_2_ cause oxidative damage in cells [30]. In the present study, IPEC-J2 cells treated with H_2_O_2_ experienced a dose- and time-dependent loss of cell viability. However, pretreatment of cells with OEO at an appropriate concentration (2.5, 5, or 10 *μ*g/mL) significantly reduced this loss of cell viability, affirmed by attenuation in LDH release. Moreover, pretreatment of cells with OEO suppressed H_2_O_2_-induced ROS and MDA production, in accordance with an inhibition of oxidative stress. This inhibition of H_2_O_2_-induced ROS generation and cell lipid peroxidation by pretreatment with OEO may occur via a direct antioxidant mechanism through free radical-scavenging activity. Polyphenolic compounds like OEO exert a wide range of antioxidant effects, acting as ROS scavengers and free radical reaction terminators [31, 32]. Pretreatment of cultured IPEC-J2 cells with OEO also significantly increased and restored intracellular GSH and SOD1 levels, possibly as a result of the antioxidative action of OEO against H_2_O_2_-induced oxidative damage.

To protect against the harmful effects of ROS, antioxidant enzymes such as CAT and SOD detoxify ROS to safe molecules [33]. SOD averts oxidative stress by catalyzing superoxide anions to H_2_O_2_ and CAT further reduces redox damage by catalyzing the reduction of H_2_O_2_ [34]. Thus, increased expression of SOD and CAT may protect against oxidative stress. The tripeptide GSH (*γ*-L-Glu-L-Cys-Gly) is the most abundant intracellular nonprotein thiol compound in mammalian cells [35]. Importantly, GSH functions as an antioxidant with a crucial role in scavenging toxic free radicals [36]. Thus, endogenous GSH is consumed during oxidative stress, resulting in a depletion of cellular GSH [37]. GSH contains an unusual peptide linkage between the *γ*-carboxyl of glutamate and the *α*-amino group of cysteine [38]. The formation of *γ*-glutamylcysteine is the first step in GSH synthesis. It is catalyzed by GCL, a heterodimeric protein composed of catalytic (GCLC) and modifier (GCLM) subunits, which are encoded by separate genes in mammals [39]. GCLC exhibits all of the catalytic activity, but its association with GCLM alters its kinetic properties to enhance GCL activity [40]. Glycine is subsequently added to c-glutamylcysteine in a second reaction that is catalyzed by glutathione synthase to form GSH [38].

In recent years, several studies have reported that plant-derived compounds activate Nrf2-dependent ARE activity and elevate the levels of cellular CAT, SOD1, and GSH, which exhibit cytoprotective effects against oxidative stress in various cells [30, 41]. The action of Nrf2 plays a key role in the adaptive response to oxidative stress and regulates ARE-driven antioxidant gene expression (including those encoding SOD1, CAT, GCLC, and GCLM) [42]. Under conditions of homeostasis, Nrf2 is retained in the cytosol, bound to a cluster of proteins that includes its cytosolic inhibitor, Keap1 [43, 44]. When stimulated, it dissociates from Keap1 and moves to the nucleus to bind with ARE to regulate the transcription of antioxidant genes [45]. Here, when we pretreated IPEC-J2 cells with OEO, Nrf2 dissociated from the inhibitory protein Keap1 and translocated to the nucleus, as indicated by a dramatic increase in nuclear Nrf2. Accumulated nuclear Nrf2 has been demonstrated to bind to the ARE and lead to transcriptional activation of antioxidant genes [46]. Our findings further confirm these molecular cascades by reporting increased ARE activity in IPEC-J2 cells treated with OEO. Activation of Nrf2 has been shown to regulate important cellular antioxidant genes, including SOD1, CAT, GCLC, and GCLM, and protect the cells from oxidative damage. These data are consistent with previous observations that plant extracts (including those from Willow bark, Ginkgo biloba, Nelumbo nucifera leaves, Piperaceae leaves, and Grape pomace) increase cellular antioxidant gene expression by increasing the levels of nuclear Nrf2 [30, 42, 47–49]. Knockdown of Nrf2 with siRNA inhibited not only OEO-induced expression of these antioxidant enzymes, but also its protective effect against oxidative stress-induced cellular damage in IPEC-J2 cells. In this study, while the increase in GCLM, GCLC, and Nrf2 mRNA expression levels and intracellular GSH caused by OEO was significantly suppressed by Nrf2 siRNA, treatment with OEO still increased GCLM, GCLC, and Nrf2 mRNA expression levels and intracellular GSH under conditions of Nrf2 suppression. However, the efficiency with which siRNAs inhibited Nrf2 was relatively low, with only 53% downregulation of Nrf2 gene expression and 47% downregulation of protein abundance. Alternatively, it has been shown that the forkhead box O (FOXO) family of transcription factors, tumor suppressor p53, and activator protein 1 (AP-1) also have major roles in preventing oxidative stress by inducing antioxidant gene expression [50–52]. Thus, Nrf2 may not be the only factor modulating oxidative stress, and OEO may modulate oxidative stress by one or more different pathways.

## 5. Conclusions

In conclusion, this study provides evidence for the involvement of OEO in protecting against oxidative stress in IPEC-J2 cells and elucidates several underlying mechanisms. Treatment of IPEC-J2 cells with OEO enhanced SOD1 and GSH expression through activation of the Nrf2/ARE pathway. This mechanism may be pivotal to its antioxidative action against H_2_O_2_-induced cell damage. ROS are essential factors in the pathogenesis of many gastrointestinal diseases. Our data support the need for further research into OEO activation of Nrf2 and the endogenous antioxidant response as a potential therapeutic approach for these diseases. OEO may indeed have broad applicability to therapies for oxidative stress-related intestinal diseases.

## Supplementary Material

Typical chromatogram of oregano essential oil components.

## Figures and Tables

**Table 1:** Chemical composition of oregano essential oil.

Components^a^	Composition%
*α*-Thujene/*α*-pinene	0.56
Camphene	0.08
*β*-Pinene	0.09
Sabinene	0.03
Myrcene	0.91
*α*-Phellandrene	0.09
*α*-Terpinene	0.50
Limonene	0.15
1,8-Cineole+*β*-phellandrene	0.07
*β*-Ocimene	0.07
*γ*-Terpinene	4.54
3-Octanone	0.07
*ρ*-Cymene	3.11
Terpinolene	0.05
3-Octanol	0.11
1-Octen-3-ol	0.22
Dimethyl styrene	0.10
Trans-Sabinene hydrate	0.14
Linalool	0.32
Cis-Sabinene hydrate	0.03
1-Terpilool	0.05
Terpinen-4-ol	0.22
Carvacrol methyl ether	0.33
*β*-Caryophyllene	1.43
Dihydrocarvone	0.09
*α*-Humulene	0.08
*α*-Terpineol	0.21
Bomeol	0.33
*β*-Bisabolene	0.71
Caryophyllene oxide	0.16
Thymol	1.90
Carvacrol	79.92
Total	99.99

^a^The dates were provided by Meritech Bioengineering Co. Ltd.
