# Supplementary material for: Oregano Essential Oil Induces SOD1 and GSH Expression through Nrf2 Activation and Alleviates Hydrogen Peroxide-Induced Oxidative Damage in IPEC-J2 Cells
Source: Oxid Med Cell Longev. 2016 Dec 26;2016:5987183. doi: 10.1155/2016/5987183 (PMC5220500; doi:10.1155/2016/5987183)
Supplement: Supplementary file 1 — Typical chromatogram of oregano essential oil components. [file 5987183.f1.pdf]

Certificate of Analysis

Contact:	Ms Songrong Qu	Report No:	MGZ-0008
Company:	Meritech Biotech	Our Ref:	MGZ-GC-008
Address:	Intl Bio-Island, Luoxuan 4th Rd	Received:	10-12-11
	Rm 405 R&D Unit A	Page No:	1 of 1
	Guangzhou China 510300		

<u>ANALYSIS OF OREGANO OIL GC-MS</u>

**Component**	**Oregano oil (MGZ-008)**
	%
α-Thujene/α-Pinene	0.56
Camphene	0.08
β-Pinene	0.09
Sabinene	0.03
Myrcene	0.91
α-Phellandrene	0.09
A-Terpinene	0.50
Limonene	0.15
1,8-Cincole+β-phellandrene	0.07
β-Ocimene	0.07
r-Terpinene	4.54
3-Ocimene	0.07
P-Cymene	3.11
Terpinoiene	0.05
3-Octanoi	0.11
1-Octen-3-ol	0.22
Dimethyl styrene	0.10
Trans-Sabinene hydrate	0.14

Linalool	0.32
cis-Sabinene hydrate	0.03
1-Terpincol	0.05
Terpinen-4-ol	0.22
Carvacrol methyl ether	0.33
B-Caryophyllene	1.43
Dihydrocarvone	0.09
α-Humulene	0.08
α-Terpineol	0.21
Borneol	0.33
β-Bisabolene	0.71
Caryophylene oxide	0.16
Thymol	1.90
Carvacrol	79.92

Signature

Songrong Qu

These results relate only to the sample(s) tested and do not guarantee the bulk of the mentioned to the equal quality.

Figure 1. Typical chromatogram of oregano essential oil components.

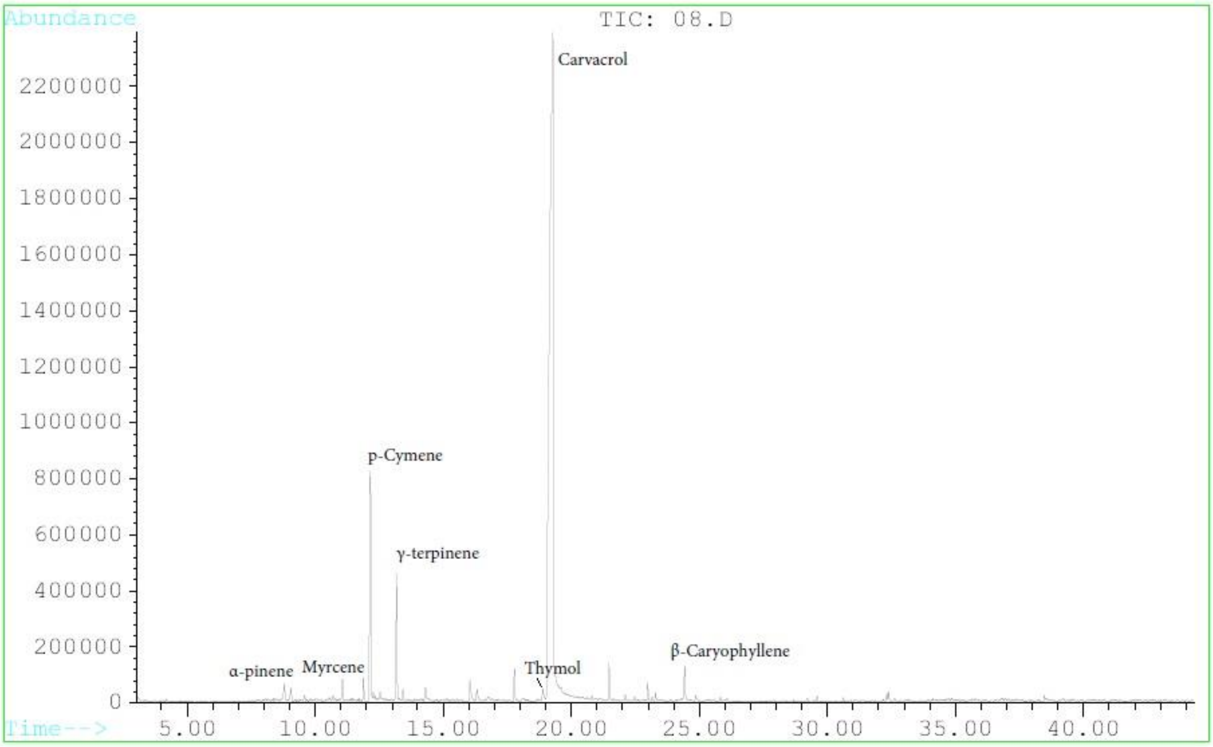

The Figure were provided by Meritech Bioengineering Co. Ltd.
